# Type 2 Diabetes in Xinjiang Uygur Autonomous Region, China

**DOI:** 10.1371/journal.pone.0035270

**Published:** 2012-04-10

**Authors:** Yi-Ning Yang, Xiang Xie, Yi-Tong Ma, Xiao-Mei Li, Zhen-Yan Fu, Xiang Ma, Ding Huang, Bang-Dang Chen, Fen Liu, Ying Huang, Cheng Liu, Ying-Ying Zheng, Gulinaer Baituola, Zi-Xiang YU, You Chen

**Affiliations:** 1 Department of Cardiology, First Affiliated Hospital of Xinjiang Medical University, Urumqi, People’s Republic, China; 2 Xinjiang Key Laboratory of Cardiovascular Disease Research, Urumqi, People’s Republic China; Fundación para la Prevención y el Control de las Enfermedades Crónicas No Transmisibles en América Latina (FunPRECAL), Argentina

## Abstract

**Background:**

The aim of this study was to estimate the prevalence and distribution of type 2 diabetes and to determine the status of type 2 diabetes awareness, treatment, and control in Xinjiang, China. Our data came from the Cardiovascular Risk Survey (CRS) study designed to investigate the prevalence and risk factors for cardiovascular diseases in Xinjiang from October 2007 to March 2010. A total of 14 122 persons (5583 Hans, 4620 Uygurs, and 3919 Kazaks) completed the survey and examination. Diabetes was defined by the American Diabetes Association 2009 criteria.

**Methodology/Principal Findings:**

Overall, 9.26% of the Han, 6.23% of the Uygur, and 3.65% of the Kazak adults aged ≥35 years had diabetes. Among diabetes patients, only 53.0% were aware of their blood glucose level, 26.7% were taking hypoglycemic agents, and 10.4% achieved blood glucose control in Han, 35.8% were aware of their blood glucose level, 7.3% were taking hypoglycemic agents, and 3.13% achieved blood glucose control in Uygur, and 23.8% were aware of their blood glucose level, 6.3% were taking hypoglycemic agents, and 1.4% achieved blood glucose control in Kazak, respectively.

**Conclusions/Significance:**

Our results indicate that diabetes is highly prevalent in Xinjiang. The percentages of those with diabetes who are aware, treated, and controlled are unacceptably low. These results underscore the urgent need to develop national strategies to improve prevention, detection, and treatment of diabetes in Xinjiang, the west China.

## Introduction

Type 2 diabetes mellitus (T2DM) is now a global health problem and a growing cause of death and disability, mainly through cardiovascular diseases [1]–[3]. The International Diabetes Federation has predicted that the number of individuals with diabetes will increase from 240 million in 2007 to 380 million in 2025 and further increase to 439 million in 2030 [4]–[5].

In China, the prevalence of diabetes increased from 1% in 1980 to 5.5% in 2001[6] with much higher rates in urban areas such as Shanghai [7], Hong Kong and Taiwan [8]. Xinjiang, a province of China, is part of the ancient Silk Road and borders eight countries including Russia, Kazakhstan, Kirghizastan, Tajikistan, Pakistan, Monogolia, India and Afghanistan. There are more than 13 ethnic groups living in this area. Among them, the Uygur people account for 46%, Han account for 40%, and Kazak account for 7%. Although the racial/ethnic disparities associated with type 2 diabetes are well known [9], there are limited data on the prevalence of diabetes among Han, Uygur, and Kazak population in Xinjiang. Awuti reported the prevalence of diabetes in Uygur population was 9.0% in 2010 [10]. Tao reported the prevalence of diabetes was 8.16% in Uygur and 1.47% in Kazak population in 2008, respectively [11]. However, they did not report the awareness, treatment, and control of diabetes in these two ethnics population. Furthermore, these two researches enrolled very small samples and did not use a province-wide population-based representative random sample. To better understand the prevalence, awareness, treatment, and control of type 2 diabetes in Han, Uygur and Kazak population in Xinjiang, an epidemiological study enrolled 14 618 participants was conducted from 2007 to 2010, which is expected to provide a comprehensive update to the results of the previous studies.

## Methods

### Ethics Statement

The present study was conducted in accordance with the Declaration of Helsinki guidelines, and written informed consent was obtained from each individual according to a protocol approved by the Ethics Committee of the First Affiliated Hospital of Xinjiang Medical University.

### Subjects

The subjects came from the Cardiovascular Risk Survey (CRS) described in the previous study [12]–[14]. Briefly, The Cardiovascular Risk Survey (CRS) study is a multiple-ethnic, community-based, cross-sectional study designed to investigate the prevalence and risk factors for cardiovascular diseases and to determine the genetic and environmental contributions to atherosclerosis, coronary artery disease (CAD) and cerebral infarction (CI) of Chinese Han, Uygur, and Kazakh population in Xinjiang of west China from October 2007 to March 2010. We used a stratified sampling method to select a representative sample of the general population of Chinese Hans, Uygurs, and Kazakhs of this area. Seven cities (Urumqi, Kelamayi, Hetian, Zhaosu, Fukang, Tulufan, and Fuhai) were chosen and, based on the government record of registered residence, one participant was randomly selected from each household. In this way, a total of 14 618 participants (5 757 Hans, 4 767 Uygurs, and 4 094 Kazakhs), were randomly selected from 26 villages of these seven cities and were invited to participate. Those whose data were incomplete were excluded. Finally, 14 122 persons (5583 Hans, 4620 Uygurs, and 3919 Kazaks) completed the survey and examination. The overall response rate was 96.61%.

We defined diabetes by using the American Diabetes Association (ADA) 2009 criteria [15] (fasting plasma glucose ≥7.0 mmol/L [≥126 mg/dL]) or self-reported current diabetes treatments in the survey. Awareness of diabetes was defined as self report of any prior diagnosis of diabetes by a health care professional among the population defined as having diabetes. Treatment of diabetes was defined as use of a prescription medication for management of diabetes at the time of the interview. Control of diabetes was defined as pharmacological treatment of diabetes associated with an average fasting GLU level <7.0 mmol/L [<126 mg/dL].

Data collection was conducted in examination centers at local hospital in the participants’ residential area. At the time of the in-person interview, a 5-mL fasting blood samples (at least fasting 8 hours) were collected with an EDTA vacutainer tube. The samples were kept in a portable Styrofoam box with ice packs (0–4°C) and were processed within 4 h. All samples were stored at −80°C immediately after processing. We measured the concentration of fasting glucose using equipment for chemical analysis (Dimension AR/AVL Clinical Chemistry System, Newark, NJ, USA) employed by the Clinical Laboratory Department of the First Affiliated Hospital of Xinjiang Medical University.

During clinic or home visits, trained research staff administered a standard questionnaire. Information on demographic characteristics-including age, gender, education, address, ethnicity, occupation, and household income-was collected. The interview included questions related to the diagnosis and treatment of diabetes. Information on the awareness of, and drug treatment and lifestyle modification for, diabetes was also obtained.

### Data Management and Statistical Analysis

All questionnaire data was double-entered and cross-validated using EpiData version 3.1 (EpiData Association, Odense, Denmark). Statistical analyses were performed in SPSS version 17.0 (SPSS Institute, Chicago, IL, USA). Continuous variables were summarized with mean and median, and categorical variables as percentages. χ^2^-test was used to explore associations in categorical data. Student's t-test was used to compare two means in numerical data. Two tailed tests were performed with the significance level at 0.05.

## Results

### Prevalence

The characteristics of study participants were shown in table 1. As shown in table 2, the prevalence of T2DM was 9.26% in Han, 6.23% in Uygur, and 3.65% in Kazak population, respectively. The overall prevalence of T2DM was the highest in Han and the lowest in Kazak among these three ethnics. In each ethnic, the overall prevalence of T2DM was higher among men than among women. For every age group up to 74 years, the prevalence of T2DM increased with age in both men and women. The prevalence of obesity in Kazak population was higher than that in Han or in Uygur population.

**Table 1:** Participant Characteristics.

	Mean±SD or No. (%)		
	Han (n=5583)	Uygur (n=4260)	Kazak (n=3919)
Age, years	52.6±12.7	50.7±13.0	48.7±11.7
Height, cm	163.2±8.5	159.8±8.5	162.7±8.7
Weight, Kg	67.2±11.9	66.1±12.9	70.6±15.1
BMI, kg/m^2^	25.1±3.5	25.8±4.4	26.6±4.8
SBP, mmHg	132.9±19.9	131.5±21.2	140.4±25.1
DBP, mmHg	85.0±15.5	80.1±14.9	88.5±19.7
Waist Circumference, cm	86.8±10.3	88.2±12.3	88.3±13.3
Hip Circumference, cm	95.8±7.4	97.7±10.0	99.6±9.7
Sex, women, n (%)	2888 (51.7)	2670 (57.8)	2025 (51.7)
Smoking, n (%)	1751 (31.4)	872 (18.9)	1398 (35.7)
Alcohol drinking, n (%)	1059 (19.0)	457 (9.9)	570 (14.5)
Diabetes, n (%)	517 (9.3)	288 (6.2)	143(3.6)
Treated diabetes, n (%)	138 (2.5)	21 (0.5)	9 (0.2)

Continuous variables are presented as mean±SD, whereas categorical variables are presented as counts and percentages.

**Table 2:** Prevalence of diabetes in adult population, aged ≥35 years, in Xinjiang, 2007–2010.

Age, y	Men			Women			Total		
	N	Diabetes [n, (%)]	Obesity [n, (%)]	N	Diabetes [n, (%)]	Obesity [n, (%)]	N	Diabetes [n, (%)]	Obesity [n, (%)]
Han ethnic									
35–44	986	64(6.49)	201(20.4)	966	25(2.59)	62(6.4)	1952	89 (4.56)	263(13.5)
45–54	644	79(12.27)	145(22.5)	676	47(6.95)	91(13.5)	1320	126 (9.55)	236(17.9)
55–64	444	37(8.33)	113(25.5)	616	73(11.85)	148(24.0)	1060	110 (10.38)	261(24.6)
65–74	465	71(15.27)	102(21.9)	530	81(15.28)	138(26.0)	995	152 (15.28)	240(24.1)
≥75	156	27(17.31)	31(19.9)	100	13(13.00)	22(22.0)	256	40 (15.63)	53(20.7)
Total	2695	278(10.32)	592(22.0)	2888	239(8.28)*	461(16.0)	5583	517(9.26)**	1053(18.9)**
Uygur ethnic									
35–44	587	26(4.43)	131(22.3)	1021	22(2.15)	279(27.3)	1608	48 (2.99)	410 (25.5)
45–54	485	28(5.77)	145(29.9)	741	45(6.07)	277(37.4)	1226	73 (5.95)	422 (34.4)
55–64	481	42(8.73)	114(23.7)	573	49(8.55)	193(33.7)	1054	91 (8.63)	307(29.1)
65–74	291	29(9.97)	71(24.4)	273	35(12.82)	84(30.8)	564	64 (11.35)	155(27.5)
≥75	106	6(5.66)	16(15.1)	62	6(9.68)	13(21.0)	168	12 (7.14)	29 (17.3)
Total	1950	131(6.72)	477(24.5)	2670	157(5.88)	846(31.7)	4620	288 (6.23)	1323(28.6)
Kazak ethnic									
35–44	777	34(4.38)	270(34.7)	871	7(0.80)	162(18.6)	1648	41 (2.49)	432 (26.2)
45–54	509	28(5.50)	207(40.7)	581	20(3.44)	249(42.9)	1090	48 (4.40)	456(41.8)
55–64	355	20(5.63)	142(40.0)	375	11(2.93)	174(46.4)	730	31 (4.25)	316(43.3)
65–74	203	12(5.91)	75(36.9)	168	8(4.76)	64(38.1)	371	20 (5.39)	139(37.5)
≥75	50	3(6.00)	18(36.0)	30	0(0.00)	9(30.0)	80	3 (3.75)	27(33.8)
Total	1894	97(5.12)	712(37.6)	2025	46(2.27)*	658(32.5)	3919	143 (3.65)	1370(35.0)

### Mean Glucose Levels

Table 3 presents gender- and ethnic-specific mean glucose values for 3 subgroups defined by diabetes status. Overall, the average glucose levels for no-diabetes subjects were 2.33, 3.30, and 3.18 mmol/L lower than the corresponding values for those with treated diabetes, and 4.75, 5.01, 5.31 mmol/L lower than the corresponding values for those with untreated diabetes in Han, Uygur, and Kazak population, respectively. In contrast, the overall levels of glucose differences between treated and untreated participants with diabetes were only 2.42, 1.71, and 2.13 mmol/L in Han, Uygur, and Kazak population, respectively.

**Table 3:** Glucose levels in adult population by diabetes status and ethnics in Xinjiang, 2007–2010.

Groups		Ethnics					
		n	Han	n	Uygur	n	Kazak
No-diabetes (Mean±SD, mmol/L)	Men	2417	5.03±0.76	1819	4.59±0.81	1797	4.98±0.77
	Women	2649	4.89±0.72	2513	4.66±0.77	1979	4.91±0.72
	Total	5066	4.96±0.74	4332	4.63±0.79	3776	4.94±0.75
Untreated diabetes (Mean±SD, mmol/L)	Men	214	9.81±4.07	122	10.05±3.96	89	9.98±4.06
	Women	165	9.58±3.28	145	9.28±2.94	45	10.80±5.86
	Total	379	9.71±3.74*#	267	9.64±3.46*#	134	10.25±4.74*#
Treated diabetes (Mean±SD, mmol/L)	Men	64	7.55±2.90	9	5.32±1.90	8	7.73±2.67
	Women	74	7.08±2.62	12	9.68±5.23	1	11.20±0.00
	Total	138	7.29±2.75**	21	7.93±4.68**	9	8.12±2.75**

### Awareness, Treatment, and Control of Diabetes

Table 4 provides the percentages of participants with diabetes who were aware of their diabetes status, who were being treated with hypoglycemic medications, and who had their diabetes controlled. Overall, in Han ethnic, 53.0% of those with diabetes were aware of their diagnosis, only 26.7% were taking prescribed medication to lower their blood glucose, and only 10.44% achieved blood glucose control; in Uygur ethnic, 35.0% of those with diabetes were aware of their diagnosis, only 7.3% were taking prescribed medication to lower their blood glucose, and only 3.13% achieved blood glucose control; and in Kazak ethnic, 23.8% of those with diabetes were aware of their diagnosis, only 6.3% were taking prescribed medication to lower their blood glucose, and only 1.4% achieved blood glucose control. Among these three ethnics, the awareness, treatment, and control of diabetes was the highest in Han ethnic but the lowest in Kazak ethnic, respectively.

**Table 4:** Percentage of persons with diabetes who are aware, treated, and controlled in adult population, aged ≥35 years, in Xinjiang, 2007–2010.

Ethnics	Men [n, (%)]				Women [n, (%)]				Total [n, (%)]		
	Awareness	Treated	Controlled		Awareness	Treated	Controlled		Awareness	Treated	Controlled
Han	136 (48.9)	64 (23.0)	23 (8.27)		138(57.7)*	74(31.0)*	31 (12.97)*		274(53.0)	138(26.7)	54 (10.44)
Uygur	49 (37.4)	9 (6.9)	7 (5.34)		54 (34.4)	12 (7.6)	2 (1.27)*		103(35.8)	21 (7.3)	9 (3.13)
Kazak	27(27.8)	8 (8.2)	2 (2.06)		7(15.2)*	1(2.2)*	0		34 (23.8)	9 (6.3)	2 (1.40)
P value	0.001	<0.001	0.083		<0.001	<0.001	<0.001		<0.001	<0.001	<0.001

More women (57.7%) were aware of their diabetes than were men (48.9%), and the treatment and control were also more common among women than among men in Han ethnic. However, more men (27.8%) were aware of their diabetes than were women (15.2%), and the treatment and control were also more common among men than among women in Kazak ethnic. Although there was not difference in awareness and treatment of diabetes between men and women in Uygur population, the control rate was higher in men (5.34%) than that in women (1.27%). Less than one fourth (23.0%) of men reported that they were taking hypoglycemic medication for their diabetes in Han, and the percentage was much lower for men in Uygur (6.9%) and in Kazak population (8.2%), respectively. Only 31.0% of Han women reported that they were being treated for their diabetes. Only 7.6% of Uygur women and 2.2% of Kazak women with diabetes reported that they were being treated for their diabetes. Furthermore, only 10.44% of Han, 3.13% of Uygur, and 1.40% of Kazak population achieved blood glucose controlled.

## Discussion

In the present study, we found a very high prevalence of diabetes but a very low rate of awareness, treatment, and control in Han, Uygur, and Kazak population in Xinjiang. Especially in Uygur and Kazak population, the percentages of those with diabetes who are aware, treated, and controlled are unacceptably low. Overall, our findings indicate that 9.26% of Han, 6.23% of Uygur, and 3.65% of Kazak people more than 35 years of age in Xinjiang have diabetes. Several previous large cross-sectional studies have reported the prevalence of diabetes in China [6], [16]–[18].

Pan et al. reported the prevalence of diabetes was 2.5% in China in 1994 [16]. Wang reported the prevalence of diabetes was 3.21% in Chinese during 1995–1997 [17]. Gu et al. reported 5.2% men and 5.8% women in China aged 35 to 74 years had diabetes in 2001–2002 [6]. Liu et al. found the prevalence of diabetes and IFG in Chinese adults was 2.7% and 4.9%, respectively, in 2002 [18]. These above studies mainly enrolled Chinese Han participants. The prevalence of diabetes in the minorities of China was incompletely investigated. Recently, Tao et al. [11] investigated the prevalence of diabetes in Uygur and Kazak population in Xinjiang and found the prevalence of diabetes was 8.16% in Uygur and 1.47% in Kazak population. The prevalence of diabetes in Uygur was higher and in Kazak was lower than that of our research, respectively. However, in their study, only 1,571 Uygur and 2,913 Kazak participants were enrolled, and these subjects can not represent the whole province population for only three regions were choosen The CRS study considered adults aged ≥35 years of Xinjiang and emphasized the geographic and economic differences between northern and southern regions. Therefore, the data from the CRS provide a comprehensive update to the results of Tao et al.

Our results indicated that the prevalence of diabetes among Uygur and among Kazak in Xinjiang was significantly lower than that among Han. For the Han ethnic, the prevalence of diabetes estimated in the present study exceeds that of many developing countries [19]–[27] and is similar to that in industrial countries [28]–[31]. The overall prevalence of diabetes in US was estimated to be 5.3% in 1976–1980 [28], 8.2% in 1999–2000 [29], and 12.6% in 2005–2006 [30]. In China, the overall prevalence of diabetes was reported 1.0% in 1980 [32], 2.5% in 1994–1995[16], and 5.5% in 2000–2001[6]. During the past ten years, no further prevalence of diabetes in China was investigated. Our results indicated that the prevalence of diabetes of Han was 9.26% during 2007–2010, which was significantly higher than that of the latest survey in China.

Furthermore, the percentage of awareness, treatment, and control of diabetes is unacceptably low in the Han, Uygur and Kazak adult population, as indicated from the present study, especially in Uygur and Kazak population. Therefore, a national diabetes education program should be established to promote community- and clinic-based blood glucose screening. Physicians must regularly check their patients’ blood glucose, and a more aggressive blood glucose-lowering goal and strategy must be utilized.

Our study has several limitations. Firstly, the CRS provided only cross-sectional data and did not permit further study of risk factors for incident diabetes. Secondly, related economic factor such as household income and social data about people’s knowledge, attitudes, and beliefs about the factors contributing to health and disease were unavailable. This limited our ability to further explore why diabetes were less prevalent among Uygur and among Kazak than among Han. Finally, in this study, only fasting plasma glucose level was used to diagnose new cases of diabetes, this fact may underestimate the prevalence of diabetes.

### Conclusion

The present study indicates that the prevalence of diabetes in Xinjiang, the west China, is very high during the past years. Furthermore, the percentage of those with diabetes who are aware, treated, and controlled is unacceptably low. A national diabetes education program should be established to promote community- and clinic-based blood glucose screening.
